# Responsive Protein Hydrogels Assembled from Spider Silk Carboxyl-Terminal Domain and Resilin Copolymers

**DOI:** 10.3390/polym10080915

**Published:** 2018-08-14

**Authors:** Fang Luo, Zhi-Gang Qian, Xiao-Xia Xia

**Affiliations:** State Key Laboratory of Microbial Metabolism, Joint International Research Laboratory of Metabolic & Developmental Sciences, School of Life Sciences and Biotechnology, Shanghai Jiao Tong University, Shanghai 200240, China; fang_luo@sjtu.edu.cn (F.L.); zgqian@sjtu.edu.cn (Z.-G.Q.)

**Keywords:** hydrogel, multistimuli-responsive, protein copolymer, genetic engineering, drug delivery

## Abstract

Responsive protein hydrogels are known to respond to target external stimuli that cause changes in their properties, attracting considerable attention for diverse applications. Here we report the design and recombinant biosynthesis of protein copolymers via genetic fusion of repeating units of resilin with spider silk carboxyl-terminal (CT) domain. The resulting copolymers were thermoresponsive in aqueous solutions, and formed reversible hydrogels at low temperatures and irreversible hydrogels at high temperatures within minutes, a peculiar dual thermogelation feature endowed by the CT domain. The incorporation of resilin blocks upshifted the temperature range of reversible gelation and hydrogel stiffness, whereas the temperature of irreversible gelation was differentially affected by the length of the resilin blocks. In addition, sodium chloride and potassium phosphate at moderate concentrations downregulated both the reversible and irreversible gelation temperatures and hydrogel mechanical properties, proving the salts as another level of control over dual thermogelation. Surprisingly, the copolymers were prone to gelate at body temperature in a time-dependent manner, and the resulting hydrogels were pH-responsive to release a highly polar model drug in vitro. The newly developed resilin-CT copolymers and the multistimuli-responsive hydrogels may be potentially useful in biomedicine, such as for drug delivery.

## 1. Introduction

Among the diverse polymer-based hydrogels, protein hydrogels have attracted considerable interest due to their advantageous features such as biocompatibility, biodegradability, and low immunogenicity [1,2,3,4]. These hydrogels are usually fabricated via physical and chemical cross-linking of soluble protein polymers to form water-insoluble three-dimensional networks. Owing to the genetic basis of sequence, molecular weight, folded structure, and stereochemistry [5,6], protein polymers thus offer substantial opportunities for the design of protein hydrogels.

Recent trends in protein hydrogel design have evolved from static to dynamic controlling systems, to yield hydrogels with desirable stimuli responsiveness [7]. Responsive protein hydrogels are playing an increasingly important role in a diverse range of applications in biotechnology and medicine [8,9,10]. They commonly undergo volume and other property changes in response to external stimuli such as temperature [11], pH [12], light [13], and mechanical frequency [14]. These unique properties of responsive protein hydrogels will not only open up new avenues for a range of experiments in biological drug delivery systems [7] and tissue engineering studies [15], but also lead to a new generation of smart biomaterials that may find applications as biosensors [16] or actuators [17].

Previously, the carboxyl-terminal (CT) domain of major ampullate spidroin 1 (MaSp1) of the spider *Nephila clavipes* (NcCT) was found to form hydrogels when a moderately concentrated solution (15%) of recombinant CT was either cooled to approximately 2 °C or heated to 65 °C [18]. Intriguingly, the gelation at low temperature was reversible, a process mainly driven by hydrogen bonding and hydrophobic interactions between the globular structures of the recombinant polypeptide. Nevertheless, the gelation at high temperature was irreversible and was speculated to be a process mainly driven by hydrophobic interactions between the partially unfolded biomacromolecules. Such a dual thermosensitive peptide domain was peculiar, because thermally induced sequential gel-sol-gel transition had scarcely been reported for either synthetic or natural biopolymers. Moreover, the CTs of spidroins of different spider species are evolutionarily conserved with respect to amino acid sequences, secondary structures, and formation of dual thermosensitive hydrogels [18]. Therefore, the CT appears to be a unique building block for *de novo* design of a family of new protein polymers and hydrogel materials with tunable physiochemical and mechanical properties. However, in-depth studies on CT-based responsive protein hydrogels are needed to fully explore its potential.

Resilin is another unique protein material that has been well recognized for its remarkable properties, such as high extensibility and exceptional resilience [19,20]. This extraordinary material is normally found in specialized regions of the cuticle of most insects [20,21]. Elvin et al. reported the first recombinant resilin-like protein, rec1-resilin, which encodes the N-terminal domain (exon 1) in native resilin (fruit fly *Drosophila melanogaster* resilin gene CG15920) comprising 18 pentadecapeptide repeats (GGRPSDSYGAPGGGN) [21]. Further physiochemical analysis revealed that rec1-resilin is an intrinsically disordered polypeptide and dominated by random coils, which exhibits dual-phase transition behavior with an upper critical solution temperature (UCST) and a lower critical solution temperature (LCST) of ~6 and 70 °C, respectively. In addition, the recombinant protein was found to display multiple-stimuli responsiveness to other triggers such as pH, ion, and light [21,22]. These unique features and advantages indicate tremendous potential of resilin consensus motifs for the development of dynamic biomaterials [23]. Indeed, resilin-like homopolymers have been biosynthesized and fabricated into fascinating biomaterials for biomedical applications due to their biomimetically high elasticity and resilience, and attractive biochemical and mechanical properties [24,25,26].

Recently, modular biopolymer designs that combine the unique properties of resilin and other structural or biologically active domains have emerged as an intriguing approach for programming biomaterials to sense and aptly respond to biomechanical demands or changes in the environment [26,27,28]. For instance, Lv et al. combined random-coil-like resilin and folded GB1 domains from the streptococcal B1 immunoglobulin-binding domain of protein G to mimic the complex molecular springs found in the muscle protein titin [26]. In another example, a polypeptide containing resilin and biologically active domains such as heparin-binding and cell-binding ligands was designed for the fabrication of cross-linked hydrogel scaffold with the capacity for mouse fibroblast cell attachment and proliferation [27]. More recently, our group designed and recombinantly synthesized a resilin-silk copolymer that was thermosensitive due to the resilin blocks and prone to self-assemble into nano- to microscale fibrils due to the silk blocks (GAGAGS), leading to the formation of cytobiocompatible protein hydrogels [28]. These examples highlight the exciting opportunities for genetically engineered protein polymers in the design and fabrication of novel dynamic hydrogel materials.

In this study, we hypothesized that the integration of resilin and spider silk CT would result in copolymers that recapitulate the dual thermoresponsive gelation behavior of CT and the multiresponsiveness and improved mechanical properties of resilin. To test the hypothesis, we first designed and biosynthesized two genetically engineered copolymers, each composed of CT and resilin consensus motifs of varying lengths. Second, thermoresponsiveness of the copolymers in solution was studied, and their thermo- and ion-sensitive gelation features were explored. Finally, gelation of the copolymers at physiologic temperature (37 °C) was examined, which resulted in hydrogels with pH-dependent release of a highly polar model drug in vitro.

## 2. Materials and Methods

### 2.1. Chemical and Materials

Ampicillin, β-mercaptoethanol, imidazole, and isopropyl-β-D-thiogalactopyranoside (IPTG) were purchased from Sangon Biotech (Shanghai, China). Tryptone and yeast extract were obtained from Oxoid (Basingstoke, Hampshire, UK). Ni-NTA agarose (catalog no. 30230) and rhodamine B (catalog no. 83689) were obtained from Qiagen (Hilden, Germany) and Sigma (St. Louis, MO, USA), respectively. Coomassie Brilliant Blue R-250 (catalog no. 161-0400) was purchased from Bio-Rad (Hercules, CA, USA). All other chemicals were of the highest purity available from commercial suppliers.

PrimeSTAR Max DNA polymerase for polymerase chain reaction (PCR) was obtained from Takara Biotechnology Co., Ltd. (Dalian, China). Restriction enzymes and T4 DNA ligase were obtained from New England Biolabs (Ipswich, MA, USA). TIANprep Mini Plasmid Kit, TIANgel Midi Purification Kit, and chemically competent cells of *E. coli* DH5α and BL21(DE3) were purchased from Tiangen Biotech Co., Ltd. (Beijing, China). Amicon Ultra-15 centrifugal filter units with Ultracel-3K membranes (3 kDa molecular weight cutoff) were obtained from Millipore (Billerica, MA, USA), and a Pierce BCA Protein Assay Kit (catalog no. 23225) was purchased from Thermo Fisher Scientific Inc. (Rockford, IL, USA).

### 2.2. Construction of Protein Expression Plasmids

Plasmid vector pET-19b (Novagen, Madison, WI, USA) was employed to construct plasmids for recombinant expression of protein polymers under the IPTG-inducible T7 promoter. First, DNA encoding the C-terminal domain of spider *N. clavipes* major ampullate spidroin 1 was amplified from a template plasmid pUC57-MaSpIC [18], using PrimeSTAR Max DNA polymerase and primers FcNdeNhe (5′-AATCATATGGCTAGCGTGGGCAGCGGCG-3′) and RcXhoSpe (5′-AATCTCGAGACTAGTGCCCAGCGCCTGATACA-3′). The PCR product was digested with restriction enzymes *Nde*I and *Xho*I, agarose gel purified, and cloned into vector pET-19b at the same sites, leading to plasmid pET19b-CT1. Next, the DNA fragment harboring 4 repeats of resilin-like sequence (GGRPSDSYGAPGGGN) was released from plasmid pET19b-R4 [28] by double digest with the enzymes *Spe*I and *Pvu*I, agarose gel purified, and then ligated with the 1.3 kb *Nhe*I–*Pvu*I fragment of pET19b-CT1. The resulting plasmid was named pET19b-R4C, which allowed recombinant biosynthesis of a protein copolymer containing 4 repetitive units of resilin and the spidroin C-terminal domain. To biosynthesize another protein copolymer containing 8 repetitive units of resilin and the spidroin C-terminal domain, plasmid pET19b-R8C was created by ligating the 5.0 kb *Spe*I–*Pvu*I fragment of pET19b-R4 with the 1.5-kb *Pvu*I–*Nhe*I fragment of pET19b-R4C. The above copolymer expression plasmids were identified by restriction digest and further confirmed by DNA sequencing.

### 2.3. Protein Expression, Purification, and Identification

The recombinant plasmids were transformed into the chemically competent cells of *E. coli* BL21 (DE3), a common microbial expression host for the pET expression system. A single colony was picked on the selective Luria−Bertani (LB) solid medium supplemented with 100 µg mL^−1^ of ampicillin, inoculated into a 15 mL tube containing 4 mL of selective liquid LB medium, and then cultured overnight at 37 °C and 220 rpm in a rotary shaker. Subsequently, 500 μL of the overnight culture was transferred into a 250 mL shake flask containing 50 mL of a minimal R/2 medium (pH 6.80) supplemented with 10 g L^−1^ of glucose as a carbon source [29]. The seed cultures, 250 mL in a total of 5 flasks, were incubated until the cell optical density at 600 nm (OD_600_) reached ~3–4, and subsequently transferred into a 5 L jar fermenter (BIOTECH-5JG-7000; Shanghai BaoXing Bio-Engineering Equipment Co. Ltd., Shanghai, China) containing 2.5 L of fresh R/2 medium. The culture temperature was initially maintained at 37 °C and pH was kept at 6.80 by adding 28% (*w*/*v*) ammonia water. The dissolved oxygen (DO) concentration was kept above 40% of air saturation by automatically increasing the agitation speed from 200 to 800 rpm and by increasing the percentage of pure oxygen when agitation at 800 rpm was insufficient to maintain the DO level. A feeding solution containing 700 g L^−1^ of glucose and 20 g L^−1^ of MgSO_4_·7H_2_O was added to the bioreactor when the culture pH increased above 6.80 because of glucose depletion. When the cell OD_600_ reached ~35–40, the temperature was downshifted to 30 °C, and IPTG was added to the fermenter at a final concentration of 1 mM. After induction at 30 °C for 6 h, the bacterial cells were harvested by centrifugation and stored at −40 °C before purification of the protein copolymers from the cell pellets.

For protein purification, the cell pellets were resuspended in 20 mM Tris-HCl buffer (pH 8.0) supplemented with 150 mM NaCl and 5 mM imidazole, and then lysed using an AH-1500 high-pressure homogenizer (ATS Engineering Ltd., Toronto, ON, Canada). The homogenate was centrifuged at 15,557 g for 20 min at 20 °C. The resulting supernatant was loaded onto an Ni-NTA agarose column that had been equilibrated with the above resuspension buffer. The column was washed and eluted with the Tris-HCl buffer supplemented with imidazole at 75 and 250 mM, respectively. The eluted proteins of interest were directly concentrated and changed into 20 mM phosphate buffer (pH 7.2) through ultrafiltration using Amicon Ultra centrifugal filter units (Millipore).

The purity of the purified protein polymers was verified with 15% sodium dodecyl sulfate polyacrylamide gel electrophoresis (SDS-PAGE) using a gel loading buffer with 1% β-mercaptoethanol, which is a reducing agent. The gels were stained with Coomassie Brilliant Blue R-250 and scanned using a Microtek Bio-5000plus Imaging Densitometer (Microtek, Shanghai, China). The protein concentrations were measured using a Pierce BCA Protein Assay Kit. All the protein samples were freshly prepared before use and maintained in the phosphate buffer (pH 7.2) unless otherwise specified. The molecular weights of the purified proteins were confirmed by using a Bruker Autoflex Speed matrix-assisted laser desorption ionization time-of-flight (MALDI-TOF) mass spectrometer (Bruker Daltonics, Billerica, MA, USA).

### 2.4. Characterization of Phase Transition

The phase transition behavior of each protein polymer was characterized by monitoring the absorbance of a protein solution at 350 nm (OD_350_) as a function of temperature on a Shimadzu UV-2600 UV-vis spectrophotometer (Shimadzu Corp., Kyoto, Japan) equipped with a constant-temperature water circulator (Model SDC-6; Scientz, Ningbo, China). Each protein at 50 mg mL^−1^ in 20 mM phosphate buffer (pH 7.2) was loaded into a Suprasil quartz micro cuvette (10 mm lightpath; Hellma, Müllheim, Germany). First, the samples were tested from 20 to 4 °C with a chilling rate of 1 °C min^−1^. Then, the temperature of the cuvettes was shifted back to 20 °C, and the moisture on the cuvette surface was cleaned up to eliminate its effect. Subsequently, OD_350_ of the samples was monitored from 20 to 85 °C with a heating rate of 1 °C min^−1^.

### 2.5. Atomic Force Microscopy

Specimens for atomic force microscopy (AFM) imaging were prepared by casting a drop (10 μL) of protein solution (1 µg mL^−1^ in 20 mM phosphate buffer, pH 7.2) onto a mica surface. The mica was then incubated at either 4, 37, or 65 °C for 10−12 h to allow drying, and then rinsed gently with Milli-Q H_2_O. The samples were subsequently allowed to dry at the respective temperatures before analysis. The AFM images were acquired in tapping mode under ambient conditions using a multimode AFM (Bruker, Dresden, Germany) with a Nanoscope IIIa scanning probe controller (Digital Instruments, Santa Barbara, CA, USA). The AFM images were collected with a scanning area of 5 × 5 μm^2^ and further analyzed with Nanoscope v5.30 software (Bruker).

### 2.6. Thermoresponsive Hydrogel Formation

Each copolymer solution (200 μL) at a protein concentration of 15% (*w*/*v*) in 20 mM phosphate buffer (pH 7.2) was transferred into a glass tube and precooled to 4 °C. The tubes were first incubated at 4 °C for 10 min, and inverted to test the formation of self-supporting hydrogels. Incubation and inversion tests were then performed at temperatures ranging from 10 to 85 °C. Pictures of the inverted tubes were taken by a Canon EOS 550D digital camera (Canon, Tokyo, Japan) and processed to the same size by using Meitu version 4.0.1.2002 software (Xiamen, China).

### 2.7. Rheological Measurements

Rheological measurements of the protein samples were performed on a stress-controlled AR-G2 rheometer (TA Instruments, New Castle, DE, USA) with a 40 mm diameter plate-on-plate geometry. After instrument calibration, 500 μL of a freshly prepared protein solution was loaded onto the bottom plate at 25 °C. The top plate was then lowered to a gap distance of 350 μm. Hydrogenated silicone oil was added to the edge of the plate to avoid dehydration. After equilibration at 2 °C for 10 min, the measurements were performed at temperatures ranging from 2 to 85 °C with a heating rate of 2 °C min^−1^. The storage modulus (*G*′) and loss modulus (*G*″) were recorded as a function of temperature at a strain of 1% and a frequency of 1 Hz. In another setup, time sweeps were carried out at 37 °C with a strain of 1% and a frequency of 1 Hz.

### 2.8. pH-Responsive Drug Release Assay

Rhodamine B was used as a model polar drug to examine drug release from the protein hydrogels in vitro. Briefly, 90 μL of either R4C or NcCT at 16.7% (*w*/*v*) in 20 mM phosphate buffer (pH 7.2) was mixed with 10 μL of rhodamine B solution at 0.5 mg mL^−1^ on 96-well cell culture plates (Nest Biotechnology Co., Ltd., Wuxi, China). The mixtures were incubated statically at 37 °C overnight to allow the formation of hydrogels. Release of rhodamine B was initiated by dropping on the hydrogel surface 200 μL of phosphate buffered saline (PBS) with pH at 7.2, 6.0, or 5.0. A 20 μL aliquot of the PBS solution was sampled from the wells at predetermined time points, and an equal volume of warmed fresh buffer at each corresponding pH was added back to the wells. Rhodamine B concentrations in the sampled solutions were quantified using an external standard calibration curve. The rhodamine B fluorescence intensities at 627 nm with excitation at 553 nm were measured on a SpectraMax M5 fluorescence microplate reader (Molecular Devices Corp., Sunnyvale, CA, USA). Data are represented as the average of 3 biological replicates with standard deviation.

### 2.9. Circular Dichroism Spectroscopy

Far-UV circular dichroism (CD) spectra were acquired using a JASCO J-815 spectropolarimeter coupled with a JASCO PTC-432S Peltier temperature controller (Tokyo, Japan). Each protein (0.2 mg mL^−1^ in 20 mM phosphate buffer, pH 7.2) was loaded into a 1 mm path length quartz cuvette and equilibrated at 2 °C for 10 min before measurements. The CD data at 222 nm were collected from 2 to 85 °C with a heating rate of 2 °C min^−1^. The CD spectra were measured 3 times and averaged. The data fitting was carried out by using Origin 9.0 software (OriginLab Corp., Northampton, MA, USA).

## 3. Results

### 3.1. Design and Biosynthesis of Spider Silk CT and Resilin Copolymers

**Figure 1:**
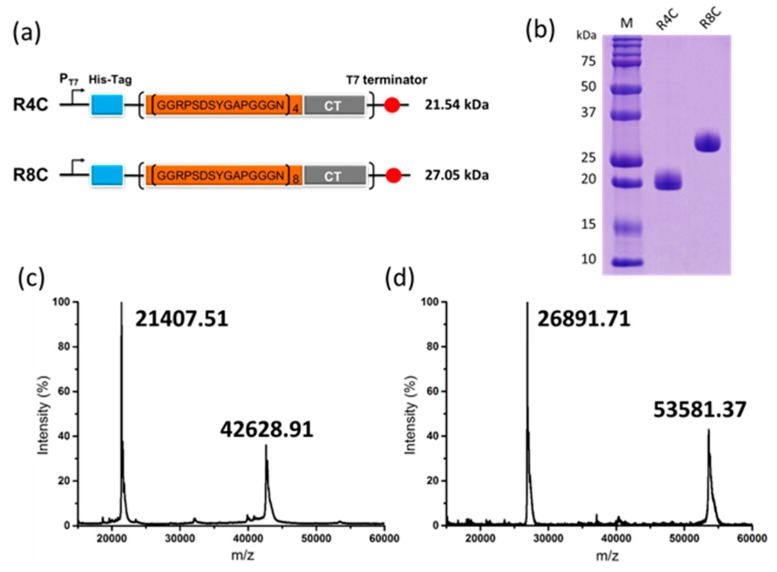
(**a**) Constructs of recombinant proteins R4C and R8C, with theoretical molecular weights calculated using the ProtParam tool (http://web.expasy.org/protparam/); (**b**) Denaturing SDS-PAGE analysis of the purified protein copolymers. MALDI-TOF mass spectra of (**c**) R4C and (**d**) R8C, which existed as monomer and homodimer under nondenaturing conditions.

In our earlier report, the dragline silk C-terminal domain of spider *N. clavipes*, which is ~110 amino acids in length, gelated at both low (~2 °C) and high (65 °C) temperatures [18]. To further explore the peculiar dual thermoresponsive gelation behavior and endow the hydrogels with responsiveness to other types of stimuli, we attempted to integrate dragline silk C-terminal domain and multiresponsive resilin. Thus, block copolymers were designed, consisting of NcCT and either four or eight resilin consensus motifs (GGRPSDSYGAPGGGN) from the first exon of the fruitfly *D. melanogaster* CG15920 gene. The two copolymers were designated as R4C and R8C, respectively (Figure 1a). The two protein copolymers were recombinantly expressed in the bacterial host *E. coli* BL21(DE3) with an N-terminal decahistidine tag, which facilitated protein purification using Ni-NTA immobilized metal-affinity chromatography.

The yields of the purified protein copolymers were in the range of 0.6−0.8 g L^−1^ of bacterial culture in a lab-scale fermenter. SDS-PAGE analysis of the proteins under denaturing conditions confirmed that the two copolymers had a purity greater than 95% (Figure 1b). The identities of the copolymers were further confirmed by MALDI-TOF mass spectrometry of the proteins under nondenaturing conditions (Figure 1c,d). It should be noted that each protein existed as a polypeptide monomer and homodimer under the native condition. Based on the mass spectra, R4C and R8C protein monomers had molecular weights at 21.41 and 26.89 kDa, respectively, which were consistent with the expected theoretical values.

### 3.2. Thermally Responsive Behavior of Copolymer Solutions

**Figure 2:**
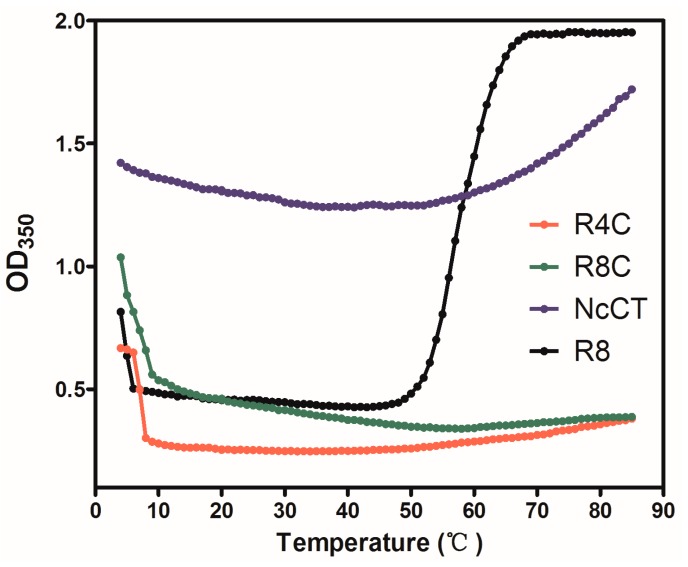
Thermally responsive behavior of the protein solutions at 50 mg mL^−1^. Turbidity profiles were recorded by monitoring OD_350_ as the protein solutions were either chilled from 20 to 4 °C or heated from 20 to 85 °C at a rate of 1 °C min^−1^. Recombinant *N. clavipes* silk C-terminal domain, NcCT [18], and a polypeptide having eight resilin consensus motifs, R8 [28], were included as controls.

To examine whether the incorporation of resilin-like blocks affected the thermoresponsive behavior, changes in optical density were monitored at 350 nm (OD_350_) of the protein solutions over a broad range of temperatures (Figure 2). Here, a resilin-like polypeptide having eight consensus motifs (R8) and NcCT, which had comparable theoretical molecular weights, were included as controls. As expected, R8 exhibited typical dual-phase transition behavior with UCST and LCST of ~4 °C and ~60 °C, respectively, which coincided well with another, larger resilin-like polypeptide (R32) in our previous study [28]. As another control, the turbidity of NcCT increased moderately upon chilling, possibly as a result of relatively weak hydrogen bonding and hydrophobic interaction between the folded protein molecules. However, a pronounced increase in turbidity was observed when the NcCT solution was heated above 60 °C, which might be due to a strong hydrophobic interaction between the exposed hydrophobic patches resulting from partial unfolding of the protein at high temperatures.

As shown in Figure 2, both R4C and R8C showed UCST-like behavior at the low temperature of ~4 °C, which was essentially the same as that of the resilin control. This result indicated that the resilin blocks in either of the two copolymers played an important role in “cold coacervation.” On the other hand, only a slight increase in solution turbidity was observed for R4C upon heating at the high temperature of ~70 °C, whereas a change in solution turbidity was almost negligible for R8C over the high temperature range. These results indicated that the coexistence of the resilin blocks and the CT domain alleviated thermal aggregation at high temperatures.

**Figure 3:**
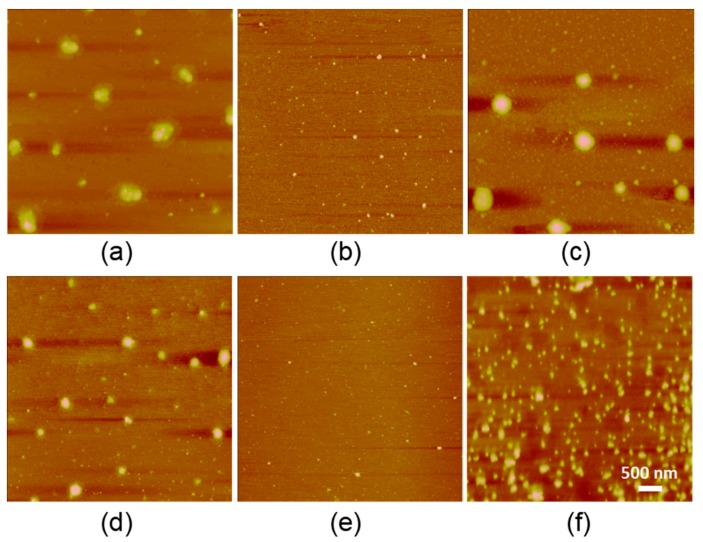
Representative atomic force microscopy (AFM) images of the nanostructures for R4C at (**a**) 4 °C; (**b**) 37 °C; and (**c**) 65 °C; and for R8C at (**d**) 4 °C; (**e**) 37 °C; and (**f**) 65 °C. Freshly prepared solution (10 μL) of each protein at a concentration of 1 μg mL^−1^ was cast on a mica surface and allowed to dry at the respective temperatures before AFM analysis.

In order to investigate the thermoresponsive self-assembly of the two copolymers, we next performed atomic force microscopy (AFM) to study the morphologies of R4C and R8C at 4, 37, and 65 °C (Figure 3). For R4C at 4 °C, some large particles with diameters of ~200−300 nm were observed among smaller particles with diameters of ~10 nm, and large particles with irregular spherical morphology appeared to be formed from the coalescence of small particles. However, R4C existed only in the form of uniform small nanoparticles with diameters of ~10 nm, reflecting no obvious aggregation of the copolymer at moderate physiologic temperature. Upon heating to 65 °C, large spherical particles with diameters of ~200−300 nm were observed again, along with large quantities of small nanoparticles with diameters of ~10 nm. Similar thermoresponsive self-assembly behaviors were also observed for R8C, except that the sizes of the physically cross-linked nanoparticles at 4 and 65 °C were smaller than those of R4C (Figure 3). Taken together, the above results demonstrate that both R4C and R8C were able to self-assemble into large nanostructures at 4 and 65 °C, and the length of the resilin blocks affected the morphologies of the thermally self-assembled nanostructures by the copolymers.

### 3.3. Thermo- and Salt-Sensitive Gelation of Copolymers

**Figure 4:**
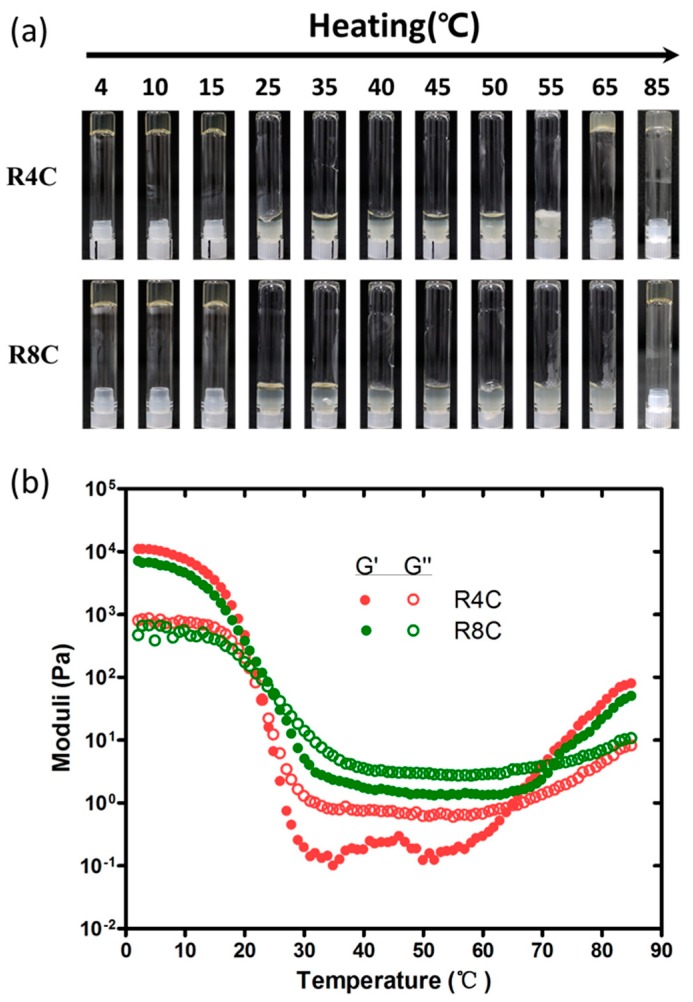
(**a**) Test of formation of hydrogels by R4C and R8C. Vials containing 15% (*w*/*v*) protein solutions were incubated for 10 min at the indicated temperatures and then inverted for image collection; (**b**) Oscillatory rheological analysis of R4C and R8C at a protein concentration of 20% (*w*/*v*). The temperature sweeps were performed with a heating rate of 2 °C min^−1^.

To test whether thermoresponsive R4C and R8C would form hydrogels, solutions of the recombinant proteins at a concentration of 15% (*w*/*v*) in phosphate buffer (pH 7.2) were incubated for 10 min at temperatures from 4 to 85 °C (Figure 4a). As expected, both R4C and R8C formed dual thermosensitive hydrogels, a peculiar feature endowed by the evolutionarily and structurally conserved spider silk CT domain, whereas the resilin control R8 did not gelate over the wide temperature range tested (data not shown). Notably, transparent reversible hydrogels of R4C and R8C were formed at low temperatures up to 15 °C, which is significantly higher than that for NcCT (~2 °C) based on the inverse gelation test in glass tubes [18]. In addition, the R4C hydrogel melted on warming and subsequently switched to an irreversible semitransparent hydrogel at 65 °C. Similarly, the R8C hydrogel melted upon warming and turned into an irreversible hydrogel at a higher temperature of 85 °C.

Next, we conducted oscillatory rheological analysis to confirm and quantify the hydrogel mechanical behavior for the two protein copolymers. To this end, the storage (*G*′) and loss (*G*″) moduli were recorded as a function of temperature at a protein concentration of 20% (*w*/*v*), which was slightly higher than that for the inverse gelation test in glass tubes. It should be noted that according to our preliminary experiments, a protein concentration of 20% was desirable to permit reliable rheological analysis, in contrast with lower protein concentrations.

As shown in Figure 4b, *G*′ of R4C was greater than *G*″ at low temperatures ranging from 4 to 23 °C, confirming hydrogel formation. In addition, the R4C hydrogel at 4 °C exhibited a storage modulus value of 11.03 kPa, which was approximately 22-fold higher than that of the NcCT hydrogel (~500 Pa) under the same conditions [18]. The storage modulus (7.14 kPa) of R8C hydrogel at 4 °C was also much higher than that of the NcCT hydrogel. Even though the R4C hydrogel was moderately stiffer than the R8C hydrogel at the chilling temperature range, the latter copolymer with the incorporation of longer resilin blocks permitted formation of the reversible low-temperature hydrogel even at 25 °C. The above results indicate that genetic fusion of resilin blocks with CT not only improved the mechanical property of the protein29 hydrogels, but also shifted the low-temperature gelation to an environmentally more benign range. This effect might be due to the resilin blocks being beneficial in enhancing hydrogen bonding and hydrophobic interactions in the copolymers, enabling the formation of reversible hydrogels with improved mechanical stiffness.

With regard to the irreversible gelation at high temperatures, the rheologic analysis revealed that R4C gelated at 65 °C, which was about 5 °C lower than that required for the formation of irreversible NcCT hydrogel [18], whereas R8C gelated at a higher temperature of 72 °C. In addition, the elastic moduli of the R4C and R8C hydrogels at 80 °C were approximately two- to threefold higher than that of the NcCT hydrogel, which was about 10 Pa in our previous study under the same rheological conditions. Again, the results prove that the resilin blocks in the copolymers affected the behavior of the irreversible gelation at high temperatures.

**Figure 5:**
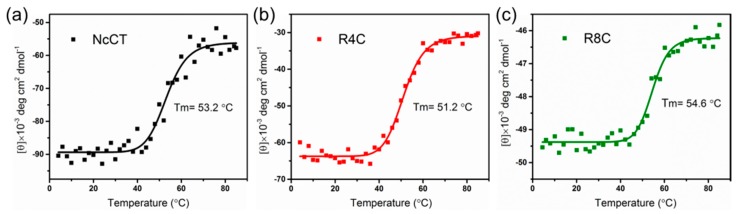
Far-UV circular dichroism (CD) unfolding data at 222 nm for (**a**) NcCT; (**b**) R4C; (**c**) R8C at a concentration of 0.2 mg mL^−1^. Solid line represents the best sigmoidal curve fit for visualization.

It was previously found that NcCT displayed a typical α-helical pattern with pronounced double minima at 208 and 222 nm at temperatures between 2 and 25 °C, and that the irreversible gelation of NcCT was ascribed to cross-linking of the exposed hydrophobic patches resulting from partial unfolding of the protein at high temperatures. To explore whether there is a link between high gelation temperature and thermostability of the copolymers, we performed far-UV circular dichroism (CD) spectroscopy to monitor α-helical unfolding of NcCT, R4C, and R8C. The CD signals at 222 nm were acquired and plotted as a function of temperature (Figure 5). As presented by the fitting curves, R4C had a melting temperature (*T*_m_) of 51.2 °C, which was lower than that for NcCT (53.2 °C), whereas a higher *T*_m_ at 54.6 °C was observed for R8C. Taken together with the aforementioned rheological data, a link was thus established between the irreversible gelation temperature and the α-helical unfolding of the proteins. Furthermore, the above results suggest that the presence of resilin blocks affected the thermal stability of the copolymers, and that the length of the resilin blocks was important in modulating the unfolding thermodynamics and the irreversible gelation behavior. It is possible that the random resilin blocks and the α-helical CT domain interacted to give rise to an overall thermodynamic and gelation behavior for the block copolymers. However, the detailed mechanism behind the differential effects of the length of resilin blocks is still obscure, and remains to be explored in future studies.

**Figure 6:**
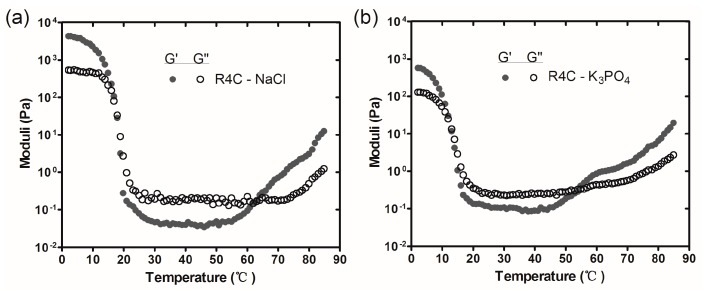
Oscillatory rheological profiles of 20% (*w*/*v*) R4C in 20 mM phosphate buffer (pH 7.2) supplemented with (**a**) 75 mM NaCl or (**b**) 75 mM K_3_PO_4_. The temperature sweeps were performed with a heating rate of 2 °C min^−1^.

Based on the fact that resilin was responsive to multiple stimuli, such as salts [30], we next examined whether gelation of the resilin-CT copolymers was affected by salts. Here, R4C was used as a model resilin-CT copolymer, and two physiologically relevant salts, sodium chloride and potassium phosphate, were included in the tests. As shown in Figure 6, dual thermosensitive gelation of R4C was observed in the presence of 75 mM NaCl or K_3_PO_4_. However, the temperatures at which R4C gelated and the mechanical properties of the hydrogels were significantly altered by the salts. For example, 20% (*w*/*v*) solution of R4C with NaCl gelated at 17 °C and 62 °C, respectively (Figure 6a), whereas the copolymer gelated at 23 °C and 65 °C in the absence of the salt (Figure 4b), indicating a downshift of both gelation temperatures. In addition, the elastic moduli of the R4C hydrogels decreased with NaCl addition. For example, a storage modulus of 4.07 kPa was observed for the R4C hydrogel with NaCl at 4 °C, whereas it was 11.03 kPa for the R4C hydrogel without NaCl at the same temperature. Notably, the effects of downshifting dual gelation temperatures and softening hydrogels were more pronounced for K_3_PO_4_ (Figure 6b). It is possible that at low temperatures, the presence of salts deteriorated the hydration layer of the protein copolymer and weakened hydrogen bonding and hydrophobic interactions between folded protein molecules, resulting in a temperature downshift in the reversible gelation and hydrogel stiffness. On the other hand, the thermally triggered protein unfolding might be favored in solutions with salts, and thus the irreversible high-temperature gelation was also downshifted.

### 3.4. Time-Dependent Gelation at 37 °C for pH-Responsive Drug Release

**Figure 7:**
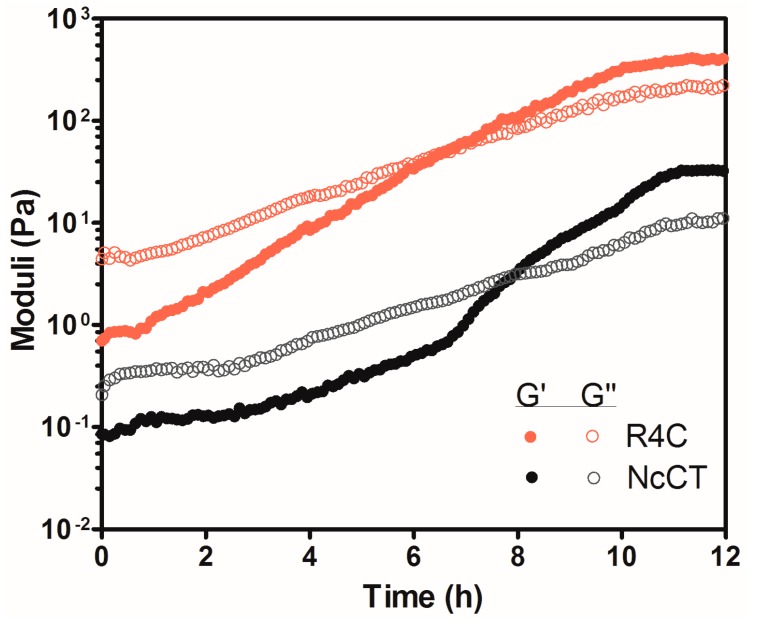
Oscillatory rheological profiles for 15% (*w*/*v*) protein solutions of R4C and NcCT as a function of time. The time sweeps were performed at 37 °C with a strain of 1% and a frequency of 1 Hz.

To examine whether R4C could gelate at physiologically relevant temperatures for potential applications, we recorded storage and loss moduli as a function of time at 37 °C (Figure 7). The rheological time sweep revealed a pronounced time dependence of *G*′ and *G*″. This behavior is typical of a polymer cross-linking process, where the intersection of *G*′ and *G*″ denotes the time point of gelation. During the initial 7 h, the values of *G*′ were lower than those of *G*″, indicating a solution state for R4C. Upon further incubation, the copolymer turned into a hydrogel, with a plateau *G*′ value at 390 Pa. As a control, NcCT gelated at ~8 h, with a significantly lower plateau *G*′ at about 33 Pa. The time-dependent gelation behavior of NcCT and R4C was intriguing, which inspired us to explore the possible application of the hydrogels (see below).

**Figure 8:**
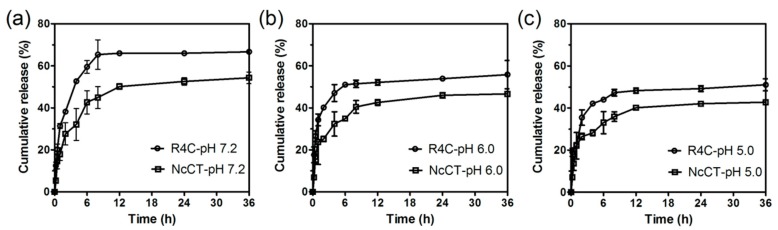
pH-dependent release of rhodamine B from R4C hydrogels in vitro. A mixture of 50 μg mL^−1^ rhodamine B and protein at 15% (*w*/*v*) was allowed to form hydrogels at 37 °C, and rhodamine B release was initiated by dropping on the hydrogel surface phosphate buffered saline (PBS) buffer with pH at (**a**) 7.2; (**b**) 6.0; and (**c**) 5.0. The hydrogels fabricated with NcCT served as controls.

Since resilin was responsive to pH, we next explored whether the above resilin-CT copolymer hydrogel allowed pH-dependent release of drugs. Here we selected as a model drug molecule rhodamine B, which is highly hydrophilic and exhibits strong fluorescent signals in aqueous solutions so that the molecule can be conveniently monitored by fluorescence spectrometry. Hence, R4C was mixed with rhodamine B, incubated overnight at 37 °C to form hydrogels, and then exposed to phosphate buffered saline at different physiologically relevant pH levels to trigger the release of rhodamine B from the hydrogels (Figure 8). For both R4C and NcCT hydrogels, there was a general trend that the cumulative release of rhodamine B was enhanced with an increase in the solution’s pH value, showing pH-sensitive behavior. For example, the R4C hydrogel released 66.0% of the loaded dye at 12 h in the pH 7.2 buffer, whereas there was only 48.3% release from the hydrogel at pH 5.0. In addition, at all three pH levels, the R4C hydrogel showed more rapid response compared with the NcCT hydrogel, indicating that the incorporation of resilin blocks in the copolymer favored pH-responsive release of the hydrophilic drug model. Taken together, the results prove that the resilin-CT copolymer hydrogels may be useful for pH-responsive delivery of drugs. Nonetheless, it should be noted that the release did not reach 100% in the above in vitro test, which might be due to the nonspecific binding between rhodamine B and protein macromolecules. It is envisioned that the hydrogel system might allow complete release of the model drug molecule in vivo due to time-dependent gradual biodegradation of the protein network.

## 4. Conclusions

In this work, we have for the first time designed and biosynthesized copolymers that are composed of the α-helical spider silk CT domain and multistimuli-responsive disordered resilin blocks. The peculiar dual thermogelation feature of CT enabled the copolymers to form a reversible hydrogel at low temperatures and an irreversible one at high temperatures within minutes. The incorporation of resilin blocks enabled reversible gelation to occur at elevated temperatures, and showed a differential effect on the irreversible gelation temperature, which depends on the length of the resilin blocks. Nonetheless, the rheological mechanical properties of both the reversible and irreversible hydrogels were consistently improved with the incorporation of resilin blocks. In addition, salts such as NaCl and K_3_PO_4_ exhibited pronounced effects on gelation temperature and hydrogel mechanical behavior, thus offering another level of control over dual thermogelation. Finally, time-dependent gelation of the copolymers within a time scale of hours at 37 °C was observed, and the resulting hydrogel exhibited pH-responsive release of a highly polar model drug in vitro. Such copolymer hydrogels with multiple-stimuli responsiveness and adjustable mechanical properties are anticipated to find wider applications in biomedicine.
